# Effect of Lung Volume on Airway Luminal Area Assessed by Computed Tomography in Chronic Obstructive Pulmonary Disease

**DOI:** 10.1371/journal.pone.0090040

**Published:** 2014-02-28

**Authors:** Kenta Kambara, Kaoruko Shimizu, Hironi Makita, Masaru Hasegawa, Katsura Nagai, Satoshi Konno, Masaharu Nishimura

**Affiliations:** 1 First Department of Internal medicine, University of Toyama, Toyama, Japan; 2 First Department of Medicine, Hokkaido University School of Medicine, Sapporo, Japan; University of California San Francisco, United States of America

## Abstract

**Background:**

Although airway luminal area (Ai) is affected by lung volume (LV), how is not precisely understood. We hypothesized that the effect of LV on Ai would differ by airway generation, lung lobe, and chronic obstructive pulmonary disease (COPD) severity.

**Methods:**

Sixty-seven subjects (15 at risk, 18, 20, and 14 for COPD stages 1, 2, and 3) underwent pulmonary function tests and computed tomography scans at full inspiration and expiration (at functional residual capacity). LV and eight selected identical airways were measured in the right lung. Ai was measured at the mid-portion of the 3^rd^, the segmental bronchus, to 6^th^ generation of the airways, leading to 32 measurements per subject.

**Results:**

The ratio of expiratory to inspiratory LV (LV E/I ratio) and Ai (Ai E/I ratio) was defined for evaluation of changes. The LV E/I ratio increased as COPD severity progressed. As the LV E/I ratio was smaller, the Ai E/I ratio was smaller at any generation among the subjects. Overall, the Ai E/I ratios were significantly smaller at the 5^th^ (61.5%) and 6^th^ generations (63.4%) and than at the 3^rd^ generation (73.6%, p<0.001 for each), and also significantly lower in the lower lobe than in the upper or middle lobe (p<0.001 for each). And, the Ai E/I ratio decreased as COPD severity progressed only when the ratio was corrected by the LV E/I ratio (at risk v.s.stage3 p<0.001, stage1 v.s.stage3 p<0.05).

**Conclusions:**

From full inspiration to expiration, the airway luminal area shrinks more at the distal airways compared with the proximal airways and in the lower lobe compared with the other lobes. Generally, the airways shrink more as COPD severity progresses, but this phenomenon becomes apparent only when lung volume change from inspiration to expiration is taken into account.

## Introduction

In bronchial asthma and chronic obstructive pulmonary disease (COPD), computed tomography has been used extensively to evaluate airway remodeling in recent years.[1]–[7] COPD is characterized by small airway remodeling and emphysema. Airway remodeling is pathologically described as increased airway smooth muscle mass and subepithelial thickening of the basement membrane in bronchial asthma.[8], [9] Thus, airway remodeling is estimated by measuring airway wall thickness and/or airway wall area corrected by airway size and/or total wall area of airways. However, validation of the measurement of such parameters has been challenged and questioned from a technical aspect, particularly when airway size is smaller.[10], [11]

Another parameter of airway dimension that can be obtained from CT data is airway luminal area (Ai), along with airway caliber. This parameter may not be suitable for an assessment of airway remodeling because airway size is changeable according to lung volume and very likely affected by the pressure balance between inside and outside the airway wall.[12] This pressure balance may be particularly important in smaller airways that lack cartilage in their walls. In other words, both intra-airway pressure determined by breathing pattern and the elastic recoil pressure of the surrounding tissue[12] would affect Ai in vivo.

However, the unique characteristics of Ai may be advantageous when examining the relationship between airway dimension and pulmonary function. Furthermore, there are some technical advantages in the measurement of Ai compared with airway wall parameters. Its assessment appears to be technically more reliable and reproducible, because the inner edge of the airway wall can be much more easily delineated than the outer edge, which would be mandatory for airway wall assessment. We often encounter serious difficulties in defining the outer edge of airways due to attachment of lung tissue and vessels, leading to potential measurement error. Indeed, our previous study showed that FEV1 % predicted is more closely correlated with Ai than airway wall parameters such as % airway wall area in patients with COPD.^5^ Furthermore, we demonstrated, using the parameter of Ai, in another study that we could quantitatively evaluate the magnitude of bronchodilation at the 3^rd^ to 6^th^ generations of airways separately, which was induced by inhaled tiotropium in patients with COPD. This approach would open the new arena because it enables us to look at any geographical difference in the effect of bronchodilation which conventional pulmonary function tests would never elucidate.[13] On the other hand, lung volume intuitively affects the size of airway, so that an assessment of Ai must be interpreted with caution when we attempt to compare Ai at different time points.

In this study, using our proprietary software, we evaluated the effect of lung volume on Ai by comparing the CT data taken at full inspiration and at expiration in COPD patients. The goal of the study was to examine the effect of lung volume change on Ai in a quantitative manner. Ederle JR et.al.[14] and Yamashiro et.al.[15] have reported positive correlations between the changes in lung volume and the changes in size of the central airways from inspiration to expiration. In this study, we attempted to extend their observations to the more distal airways and hypothesized that the effect of lung volume on Ai might differ by airway generation, lung lobe, and/or spirometric COPD severity.

## Methods

### Subjects

The subjects were 61 male and 6 female patients with clinically diagnosed COPD who participated in the Hokkaido COPD cohort study[16], [17] and agreed to have CT scans twice on one occasion. Based on the post-bronchodilator FEV1 (forced expiratory volume in 1 sec) data, the patients were diagnosed according to the GOLD criteria updated 2003[18] as: COPD at risk, 15 patients; Stage 1, 18 patients; Stage 2, 20 patients; and Stage 3, 14 patients. There were no marked physical differences, such as height and body weight, among the groups.

### Study protocol

All subjects were patients who participated in Hokkaido University Hospital. They underwent CT scans and lung function tests on a single day, except for some who attended twice within an interval of ≤1 week. Prior to the CT scans, the subjects were carefully instructed by a radiologist how to hold their breath by recorded voice instructions at deep inspiration and at relaxed expiration. This study was conducted in accordance with the amended Declaration of Helsinki. The Health Authority Research Ethics Committee of Hokkaido University School of Medicine approved the protocol as part of the Hokkaido COPD cohort study, and written, informed consent was obtained from all patients.

### Pulmonary function tests

A rolling seal type of spirometer CHESTAC-33 (CHEST M.I., Inc., Tokyo, Japan) was used. The results of pulmonary function tests met the requirements of the Japanese Respiratory Society guideline,[19] which are similar to those of the American Thoracic Society (ATS). Acceptable maneuvers were defined as those with peak expiratory flow within 10% of the maximum observed, a rapid start, absence of major flow fluctuations, and adequate expiration time. Reproducible maneuvers agreed within 200 mL of the larger FEV1. The FEV1 and forced vital capacity (FVC) values taken to characterize each participant were the maximum results obtained from acceptable maneuvers. Forced expiratory volume in 1 second (FEV1) and forced vital capacity (FVC) were expressed as percentages of predicted values according to the prediction equations of the Japanese Respiratory Society. Lung volumes (total lung capacity (TLC), functional residual capacity (FRC), and residual volume (RV)) were measured by the helium closed circuit method. Lung volumes were expressed as percentages of predicted values according to the prediction equations of Nishida.[20]

### CT Data scanning and image analysis

CT scans were performed using a multidetector-row spiral CT scanner with four detector arrays (SOMATOME plus Volume Zoom; Siemens, Berlin, Germany). CT scans were acquired with the following parameters: 120–140 kVp, 75–350 mA, 4 detector ×1 mm collimation, 1.25 mm thickness and helical pitch 7, reconstruction filter, kernel B30f, FOV 280–340×280–340 mm. In this study, the entire lung of each patient was scanned in the supine position. All CT raw data sets were reconstructed to voxel data using both soft-tissue and bone algorithms. The length of the 1-voxel side was 0.625 mm or around this value. Raw data was transferred to the workstation, and then reconstructed into three-dimensional chest images (Virtual place Fujin raijin 310, AZE Ltd., Tokyo, Japan). The detailed process of CT data acquisition and reconstruction has been described previously.[13], [21], [22] A segmental bronchus is first defined as the 3^rd^ generation of bronchi, after which one proceeds peripherally, using the longitudinal image and the short axis image simultaneously and searching for any bifurcation around the entire circumference.

At each bifurcation, in general, one bronchus was randomly selected. If the image of the bronchus was poor or it was obstructed, then the other bronchus, up to the 6^th^ generation, was selected. It was possible to compare the same sites of identical bronchi in two respiratory phases in a given subject because we use two screens that allow simultaneous assessment of dual images of inspiration and expiration.

Total lung volume (LV) on CT measurement was also calculated using the same software. In short, the whole lung containing airways (A) was extracted from the 3D image of the thorax, resulting in deletion of the heart and major vessels in the lungs. Then, the bronchial skeleton (B) was extracted from the whole lung, resulting in the lung consisting of parenchyma without either major vessels or proximal bronchial trees. LV was defined as (A)–(B).

### Data analysis

Eight bronchi were selected in the right lung: apical (B1), posterior (B2), and anterior (B3) of the upper lobe; lateral (B4) and medial (B5) of the middle lobe; and anterior basal (B8), lateral basal (B9), and posterior basal (B10) of the lower lobe. Then, Ai was measured at the midpoint between bifurcations, from the 3^rd^ to 6^th^ generation of each airway, leading to a total of 32 measurement sites per subject; the averages per generation and per lobe were calculated for the analysis. The ratio of expiratory to inspiratory LV (LV E/I ratio) and Ai (Ai E/I ratio) were defined for evaluation of changes in LV and Ai. If the LV E/I ratio was 70%, this means that the subject exhaled 30% of the inspiratory LV during expiration. To evaluate the effect of lung volume on Ai according to COPD severity, we examined the Ai E/I ratio itself and also the Ai E/I ratio corrected by lung volume change from inspiration to expiration in each subject, that is, the Ai E/I ratio divided by LV E/I ratio. This is because LV E/I ratio was highly variable according to COPD severity. All measurements were performed by one of the authors (K.K.), who was blinded to all other subject information.

### Statistical analysis

All statistical computations were performed with a statistical software package(JMP for Windows, version 8 and RX 64 3.0.0). Results are expressed as means±SD for the subjects' characteristics and the results of pulmonary function tests and as means±SEM for comparison of means of any CT parameters. Linear regression analysis was used to evaluate the relationship between LV data at expiration measured by CT and FRC values physiologically measured and the relationship between Ai and LV changes from the inspiratory to the expiratory phase. One way analysis of variance of LV E/I ratio and Ai E/I ratio among GOLD stage was done, using Tukey's honestly significant difference test. Freidman test was used for the comparison of Ai E/I ratio for the generation and for the lobe. A value of p<0.05 was considered significant.

## Results

The patients' characteristics and the results of pulmonary function testing are shown in Tables 1 and 2.

**Table 1:** Characteristics of the subjects.

Subjects	Median	Range	Mean	SD
Age (yr)	71	48–85	68	8
Height (cm)	164	149–176	164	6
Weight (kg)	61	42–92	63	11
Smoking (pack-years)	57	21–174	65	30

The subjects were 61 males and 61 females.

**Table 2:** Results of pulmonary function tests.

Stages	at risk	1	2	3	all
N	15	18	20	14	67
postFEV1[L]	2.67±0.47	2.67±0.54	1.69±0.39	1.04±0.17	2.04±0.79
post%FEV1[%]	96.1±11.1	93.2±11.3	64.0±8.8	38.4±6.1	73.7±24.5
postFEV1/FVC[%]	74.4±2.6	62.2±5.7	51.2±9.5	34.7±6.1	55.9±15.3
TLC[L]	5.71±0.79	6.51±1.15	5.94±0.61	6.75±1.08	6.21±0.99
FRC[L]	3.30±0.69	3.89±0.75	3.70±0.60	4.84±0.89	3.90±0.89
RV[L]	2.13±0.48	2.37±0.45	2.65±0.49	3.87±0.76	2.71±0.82
RV/TLC[%]	35.3±3.2	37.8±5.8	44.8±1.2	57.1±5.5	43.3±9.5

Definition of abbreviations: FVC = forced vital capacity, FEV1 = forced expiratory volume 1s, post =  post-inhalation of bronchodilator inhalation (mean±SD).

### LV measurements

**Figure 1:**
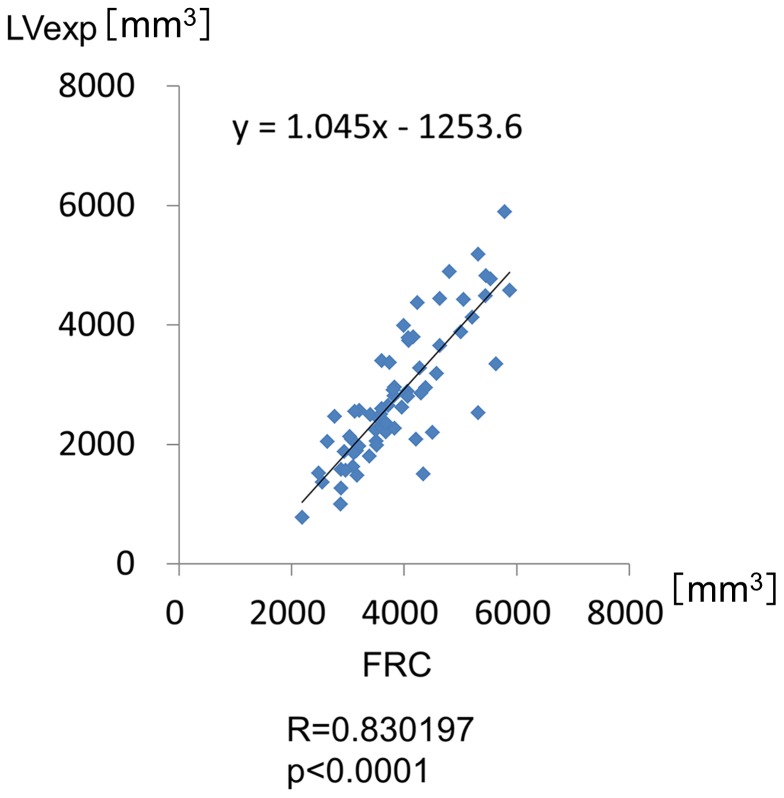
Comparison of the lung volume at expiration with the level of FRC. Comparison of the lung volume at expiration, which was calculated by CT data, with the level of FRC, which was measured by the helium closed circuit method. The lung volumes measured by the two methods are well-correlated (R = 0.83, p<0.001). LV exp: lung volume at expiration.

**Figure 2:**
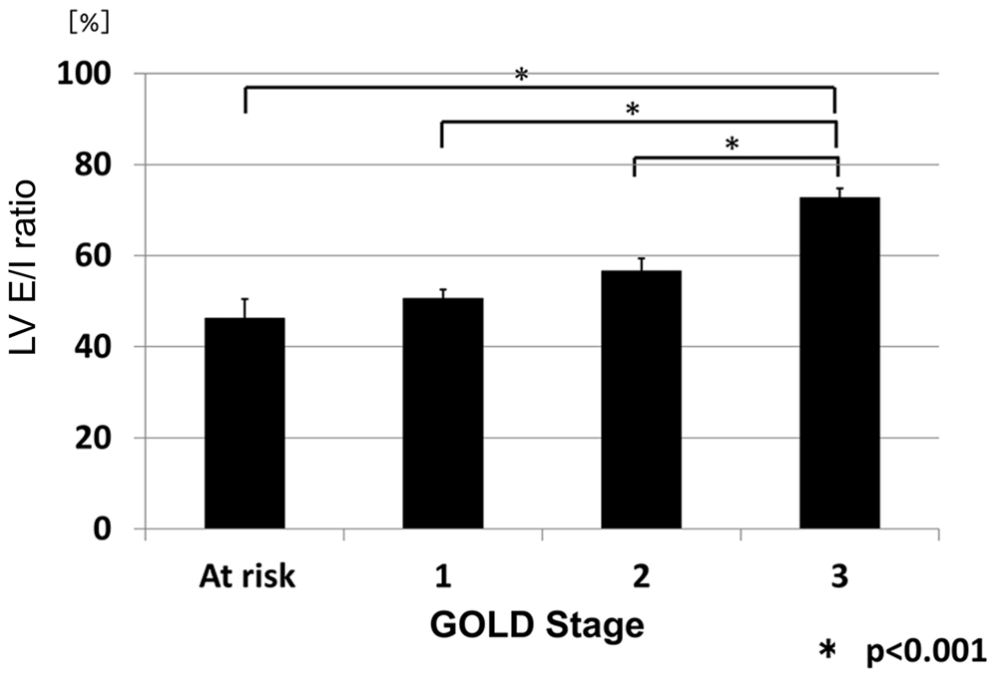
LV E/I ratio among the subjects. The LV E/I ratios were 46.2%±4.3% in subjects at risk, 50.5%±2.3% at Stage 1, 56.6%±2.8% at Stage 2, and 72.7%±2.1% at Stage 3 (p<0.001 at Stage 3 compared with other Stages). Expiration levels differed depending on the severity of airflow limitation.

It was presumed that the lung volumes at full inspiration and expiration on CT would be highly varied among the subjects because they were COPD patients with various degrees of airflow limitation. Therefore, the lung volume at expiration, which was calculated by CT data, was first compared with the level of FRC, which was measured by the helium closed circuit method. As expected, the lung volumes measured by the two methods were well-correlated (R = 0.83, p<0.001; Figure 1), which indicated that the lung volume at expiration when CT was taken would roughly represent the FRC level of the subjects. The LV E/I ratio was then calculated from CT data. The LV E/I ratio increased as the COPD stage progressed: 46.2%±4.3%(SEM) in the subjects at risk, 50.5%±2.3% at Stage 1, 56.6%±2.8% at Stage 2, and 72.7%±2.1% at Stage 3 (p<0.001 at Stage 3 compared with the other Stages; Figure 2, Table 3).

**Table 3:** Results of Lung Volume and Airway luminal area measurements.

LV E/I ratio (*p<0.01v.s. at risk)					
Stages	at risk	1	2	3	all
LV E/I ratio	46.2±4.3	50.5±2.0*	56.6±2.8*	72.7±2.1*	56.0±1.8

Ai E/I ratio (*p<0.01, **p<0.001 v.s. 3rd generation, †p<0.01, ††p<0.001 v.s. 4th generation)					
Stages	at risk	1	2	3	all
3^rd^ generation	73.0±3.3	73.8±2.6	74.2±2.1	79.8±2.5	75.0±1.3
4^th^ generation	62.1±3.6	65.8±3.2	68.9±2.7	70.5±3.6	66.9±1.6**
5^th^ generation	55.2±3.6**	57.9±3.0**†	61.6±2.5**†	64.5±3.9*	60.0±1.6**††
6^th^ generation	54.7±3.2**	59.7±3.2**†	60.5±2.6**	62.8±2.6**	59.5±1.5**††
	**Upper Lobe**		**Middle Lobe**		**Lower Lobe**
all	74.5±1.4§		74.0±1.4§		58.7±1.7

§ p<0.001v.s. lower lobe.

Definition of abbreviations: LV =  lung volume, Ai = airway luminal area, LV E/I ratio =  The ratio of expiratory to inspiratory LV, Ai E/I ratio =  The ratio of expiratory to inspiratory Ai (mean±SEM).

### Ai measurements

**Figure 3:**
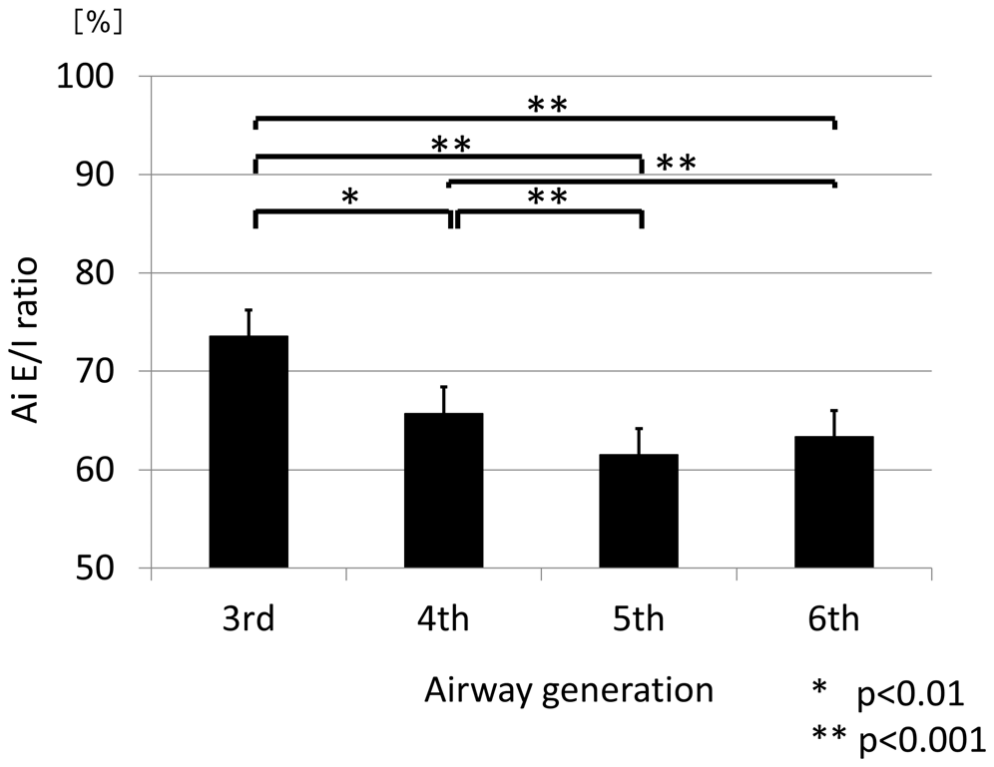
Ai E/I ratio from the 3^rd^ to the 6^th^ generation. The mean values of Ai E/I ratio of the distal airways at the 5^th^ and 6^th^ generations were significantly smaller than those of the proximal airways at the 3^rd^ and 4^th^ generations (p<0.001).

**Figure 4:**
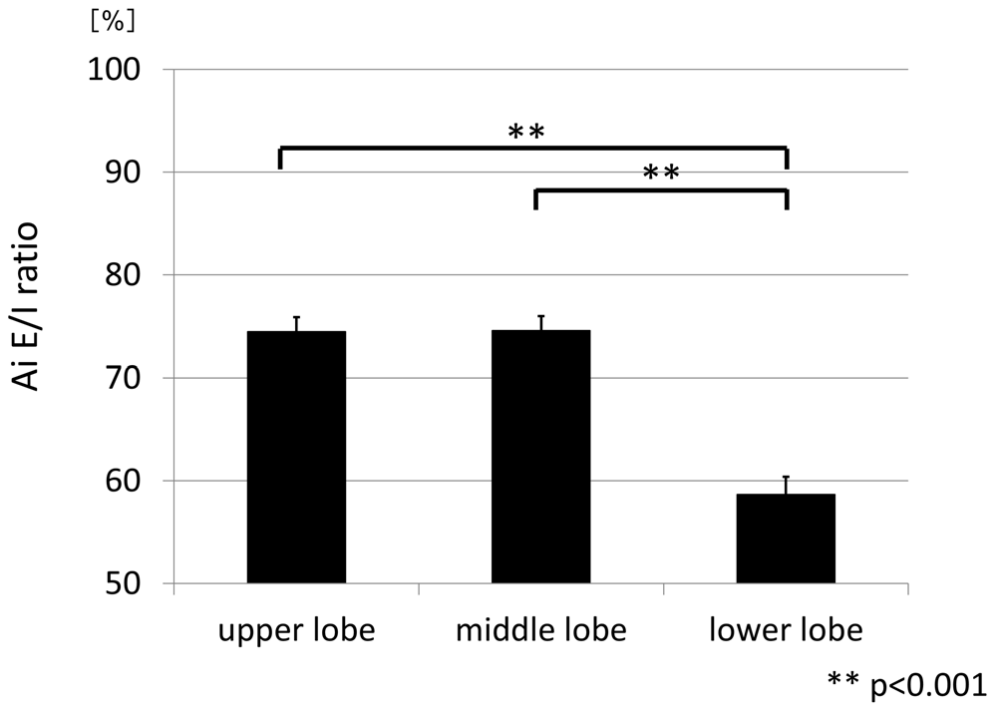
Ai E/I ratio of upper, middle and lower lobes. The mean Ai E/I ratio was significantly smaller in the lower lobe than in both the upper or middle lobes (p<0.001).

**Figure 5:**
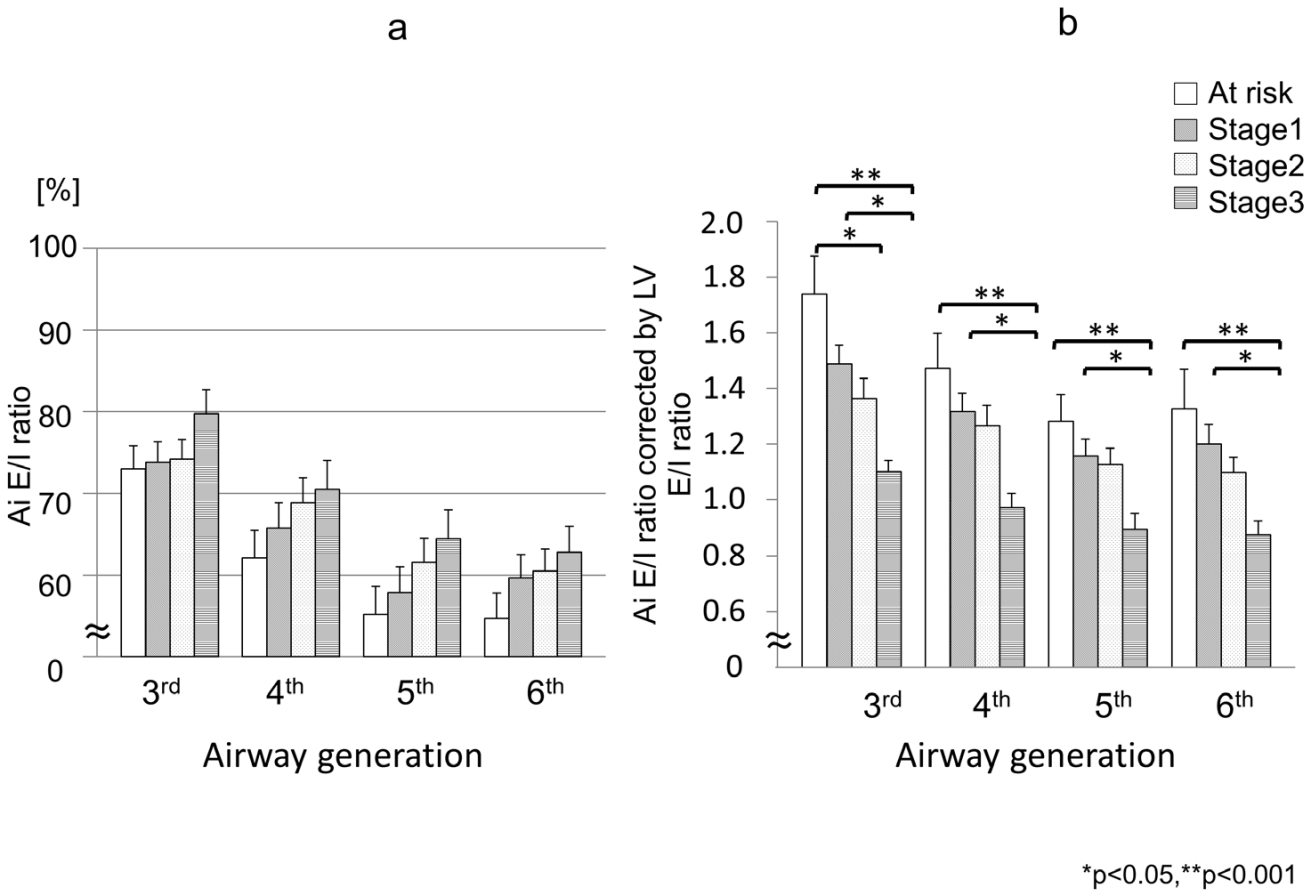
(**a**) Ai E/I ratio from the 3^rd^ to the 6^th^ generation compared according to the spirometric COPD stage. There were no significant differences in the Ai E/I ratio at any of the 3^rd^ to 6^th^ generations of the airways when compared according to the spirometric COPD stage. Rather, Ai E/I ratio of more severe COPD subjects tended to be higher comparing with mild COPD subjects. (**b**) Ai E/I ratio corrected by lung volume change from inspiration to expiration (LV E/I ratio) from the 3rd to the 6th generation compared according to the spirometric COPD stage. Ai E/I ratio corrected by lung volume change from inspiration to expiration (LV E/I ratio) was significantly different among the groups according to COPD severity.

The hypothesis that the Ai E/I ratio would differ by the generation of the airways and/or by the lobe where the airways were located was then examined, using the data from all subjects. The mean Ai E/I ratio was 73.6%±1.3% (SEM) at the 3^rd^, 65.7%±1.5% at the 4^th^, 61.5%±1.5% at the 5^th^, and 63.4%±1.4% at the 6^th^ generation. Thus, the mean Ai E/I ratios of the distal airways at the 5^th^ and 6^th^ generations were significantly smaller than those of the proximal airways at the 3^rd^ and 4^th^ generations (p<0.001 for each) (Figure 3, Table 3). The same parameters were then examined by lobe. The mean Ai E/I ratio was significantly smaller in the lower lobe than in the upper or middle lobes (p<0.001) (Figure 4, Table 3). There were no statistically significant differences in the mean Ai E/I ratio at any of the 3^rd^ to 6^th^ generations of the airways according to the spirometric COPD stage (Figure 5a, Table 3); however, if the Ai E/I ratio was corrected by the LV E/I ratio in each subject, it became smaller as the spirometric COPD severity progressed. (Figure 5b) This is because the LV E/I ratio was larger (less volume change from inspiration to expiration) as the COPD stage progressed, so that the magnitude of Ai change was seemingly smaller. In other words, the airways actually shrink more in advanced COPD from inspiration to expiration if corrected by volume change; however, it was likely to be masked by the smaller change in lung volume without such correction.

### Correlations between changes in LV and Ai

**Figure 6:**
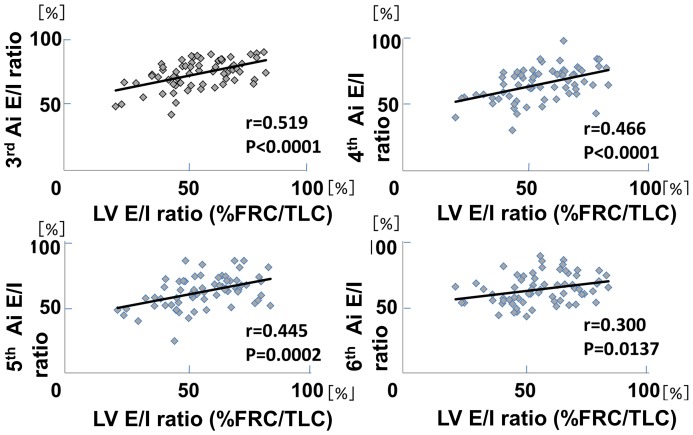
Comparisons of Ai E/I ratio with LV E/I ratio(%FRC/TLC) from the 3^rd^ to the 6^th^ generation. There were significant positive correlations between the ratio of Ai and that of LV at any generation. X-axis indicates % FRC/TLC expressed as LV E/I ratio, and Y-axis indicates Ai E/I ratio. Each dot represents the data of individual subject and thus the relation between two variables indicates the relationship, not in an individual, but among the subjects, whose % FRC/TLC was so variable, dependent on COPD stages.

The relationship between the LV E/I ratio and the Ai E/I ratio at the 3^rd^ to 6^th^ generation of the airways was next examined. Figure 6 demonstrated the relationship of the two variables, not in a single subject, but among the subjects who exhibited variable levels of LV E/I ratio based on COPD stages. The results clearly indicated that Ai E/I ratios were significantly smaller as the LV E/I ratios were smaller at any generation of the airways.

## Discussion

In this study, we first confirmed that the lung volume at expiration measured by CT was significantly well-correlated with the FRC level physiologically measured on the same day. This is very important for this study because the level of expiration could be highly variable in COPD. We then demonstrated that the change in Ai from deep inspiration to expiration differed significantly according to airway generation and also according to the lobe of the lung where the airways are located. In other words, the airway caliber shrinks more at the distal airways than the proximal airways in the 3^rd^ to 6^th^ generations and in the lower lobe compared with the upper or middle lobe when the subjects exhale from full inspiration to expiration. These data clearly indicate that we must always consider lung volume not only when assessing emphysema by lung densitometry,[23]–[29] but also when assessing Ai for comparison in any observational cohort studies and/or with any pharmacological interventions. Additionally, we found that there were significant correlations between the Ai E/I ratios and the LV E/I ratios at any of the 3^rd^ to 6^th^ generation of the airways among the subjects who exhibited variable levels of LV E/I ratio, depending on spirometric COPD stages. Finally, we demonstrated that the airways shrink more as COPD severity progresses, but this phenomenon becomes apparent only when lung volume change from full inspiration to expiration is taken into account.

It is important to note that the CT scans were conducted while the subjects were breath-holding both at full inspiration and at relaxed expiration. Several points must be considered when interpreting the present data. Firstly, dynamic Ai changes during breathing were not observed. Either inspiration or expiration may lead to dynamic pressure changes both inside and outside of the airway wall, thus potentially causing dynamic Ai changes during breathing. This may be particularly important when considering the effects of airway generation and spirometric COPD stage on Ai. Secondly, the level of expiration might vary in any individual even in the same clinical setting. In this study, prior to CT scans being taken, the subjects were carefully instructed by a radiologist how to hold their breath by recorded voice instructions at deep inspiration and at relaxed expiration. There was a significant and good correlation between LV at expiration assessed by CT and FRC measured by the helium closed circuit method, thus indicating that LV at expiration when CT scans were taken roughly represented the level of FRC in this study.

We have demonstrated that there were significant correlations between Ai E/I ratio and LV E/I ratio from the 3^rd^ to the 6^th^ generation in Figure 6, which extended the results of the previous studies showing the positive correlations between Ai E/I ratio and LV E/I ratio of the central airways.[14], [15] However, it must be noted that each dot represents the data of individual subject in Figure 6 and thus the relation between two variables indicates the relationship, not in an individual, but among the subjects whose expiration levels were so different. That might be the reason why the trend line does not go through the point (100%, 100%), which should be the case in an individual data. Quite interestingly and importantly, the slope of correlation coefficients between LV E/I ratio and Ai E/I ratio appears to get more flat as the airways go from the 3rd to the 6^th^ generation. This fascinating phenomenon may indicate the influence on COPD (the degree of airflow limitation) may be different, in reality, in the airway generations in terms of the effect of lung volume change on Ai.

The effect of inspiration level on the CT assessment of pulmonary emphysema severity has been studied extensively.[23]–[29] On the other hand, attention has been paid only recently to the assessment of airway dimensions at inspiration and expiration. Matsuoka et al.[30] demonstrated that the severity of airflow limitation assessed by pulmonary function tests was better correlated with airway caliber at expiration compared with at inspiration at the 3^rd^ to 5^th^ generations of three bronchi in 50 subjects with COPD, whose spirometric data was similar to those of our current study. In this study, correlation coefficients between FEV1 % predicted and the mean Ai at the 3rd to the 6th generation were 0.385 to 0.439 at inspiration (p<0.01, Figure S1) and 0.280 to 0.318 at expiration (p<0.05, Figure S2), which seems to be opposite to the results of Matsuoka et al since the correlation between FEV1 % predicted and Ai was apparently better at inspiration rather than at expiration in the current study. The Ai E/I ratios in their study were much smaller, 63%±13% (mean±SD), 60%±19%, and 45%±15% at the 3^rd^, 4^th^, and 5^th^ generation, compared with 73.6%±1.3% (mean±SEM), 65.7%±1.5%, and 61.5%±1.5%, respectively, in the current study. These marked differences existed despite similar spirometric data on average in the two study populations. Therefore, we speculate that the subjects were forced to exhale deeper in their study at expiration, particularly in more severe COPD, when expiratory CT was taken. Anyhow, when we take expiratory CT scans, the level of expiration would be vitally important for later analysis of airway calibers and even comparison could not be possible between the studies unless expiration level was carefully monitored. More recently, Bakker et al.[31] reported that the airway luminal areas of the 3^rd^ generation of the right apical and bilateral basal segmental bronchi were actually dependent on inspiration level in 44 subjects with COPD with alpha-1 antitrypsin deficiency, and the distensibility, defined as the difference in airway luminal area from FRC to TLC levels divided by the corresponding lung volume change, was different between the upper lobe and lower lobe, which is concordant with the present result. In the current study, their observations were further extended, as more accurate figures on the effect of lung volume from TLC to FRC on airway luminal area were provided per airway generation. The current study suggests that lung volume particularly at relaxed expiration varies highly among subjects, so that the effect of lung volume should be carefully monitored in such studies that deal with airway calibers and/or luminal area.

In contrast with examining the effect of lung volume on Ai of the airways in this study, the concept of airway distensibility has long been explored, in asthma[32]–[34] and/or COPD research, from the standpoint of airway remodeling. Brown et al.[32] failed to demonstrate a defect in the distensibility of the asthmatic airways; Castagnaro et al. and Johns et al. reported that airway distensibility might be less in bronchial asthma patients than in healthy controls.[33], [34] Airway distensibility has recently been examined in COPD patients. Scichilone et al. reported that loss of the effect of deep inspiration is strongly associated by COPD severity.[35] Diaz et al.[36] hypothesized that the airway caliber would be affected by the extent of emphysema and examined the distensibility, defined as the ratio of absolute change in airway inner diameter to the cube root of absolute change in lung volume from relaxed exhalation to full inflation (Δd/^3^√ΔLV). They found that airway distensibility was smaller in those with emphysema-predominant COPD compared with those with airway-predominant COPD. They speculated that airway-parenchymal interdependence might be impaired in emphysema-predominant COPD, thus reducing airway distensibility. Distensibility was not examined in the current study as the interest was in the effect of the change in lung volume on Ai from inspiration to expiration.

There were a couple of limitations in this study. First, since the subjects were mostly male, a potential sex-related bias was not explored. Second, lung volume was measured as a whole, but not per lobe. The finding in this study that the airway shrinks more in the lower lobe compared with the upper or middle lobe may simply reflect that the change in lung volume differs depending on lobe when the subjects exhale. Finally, since only one bronchus was randomly selected at each bifurcation, one cannot be sure that this reflects the whole picture of all airways. It is highly likely that the effect of lung volume on airway luminal area may differ depending on the nature of airway inflammation and remodeling; thus, heterogeneity must be taken into account.

In conclusion, we, in the present study, quantitatively and precisely examined the effect of lung volume change on airway luminal area in patients with COPD. In particular, we demonstrated that the lung volume effect on the Ai E/I ratio from full inspiration to relaxed expiration is greater at the distal airways and in the lower lobe of the lung in a given subject. Finally, we demonstrated that the airways shrink more as COPD severity progresses, but this phenomenon becomes apparent only when the Ai E/I ratio is corrected by lung volume change from full inspiration to expiration.

## Supporting Information

Figure S1 **Relationship of pulmonary function parameter (FEV1 %predicted) with airway luminal area (Ai) at full inspiration.** The relationships of FEV1 %predicted with the mean Ai at the 3rd to the 6th generations in all subjects at full inspiration are shown. See text if one wishes to know how the mean Ai at each generation was calculated and how CT was taken at full inspiration and expiration. There were significant correlations between FEV1 %predicted and the mean Ai at the 3^rd^ to the 6^th^ generations at any generation.
(TIF)

Figure S2 **Relationship of pulmonary function parameter (FEV1 %predicted) with airway luminal area (Ai) at expiration.** The relationships of FEV1 %predicted with the mean Ai at the 3rd to the 6th generations in all subjects at expiration (at functional residual capacity) are shown. Although statistically significant at any generation, the correlation coefficients were evidently better for the data obtained at full inspiration compared with those at expiration.
(TIF)
